# Are smoking and chlamydial infection risk factors for CIN? Different results after adjustment for HPV DNA and antibodies

**DOI:** 10.1038/sj.bjc.6601220

**Published:** 2003-08-26

**Authors:** K Matsumoto, T Yasugi, A Oki, H Hoshiai, Y Taketani, T Kawana, H Yoshikawa

**Affiliations:** 1 Department of Obstetrics and Gynecology, University of Tokyo, Tokyo 113-8655, Japan; 2 Department of Obstetrics and Gynecology, Institute of Clinical Medicine, University of Tsukuba, Tsukuba 305-8575, Japan; 3 Department of Obstetrics and Gynecology, Kinki University School of Medicine, Osaka 589-8511, Japan; 4 Department of Obstetrics and Gynecology, Mizonokuchi Hospital, Teikyo University, Kawasaki 213-8507, Japan

**Keywords:** HPV, CIN, risk factors, smoking, *Chlamydia trachomatis*, case–control study

## Abstract

To identify the risk factors for cervical intraepithelial neoplasia (CIN), we reanalysed the data from our previous case–control study by adjusting for human papillomavirus (HPV) antibodies. Unlike our previous study based only on HPV DNA, smoking and *Chlamydia trachomatis* infection were revealed as significant risk factors for CIN after adjustment for HPV antibodies.

Infections by oncogenic human papillomaviruses (HPVs) are established as a major risk factor for cervical intraepithelial neoplasia (CIN) and invasive cervical cancer (ICC). However, only a fraction of infected women develop cervical cancer, suggesting the involvement of additional cofactors in cervical carcinogenesis. In numerous case–control studies various epidemiological factors have been suggested as relevant to CIN and ICC, although the results from these studies have not been entirely consistent (Schiffman and Brinton, 1995). In epidemiological studies of risk factors for CIN and ICC, it is of great importance to allow for the strong effect of HPV infections. To determine HPV exposure among cases and controls, most studies have used HPV DNA testing, while recent studies have employed HPV capsid serology. However, it has not been determined whether the different measurements of HPV exposure can affect conclusions from case–control studies.

To address this issue, we reanalysed the data from our previous case–control study of CIN by adjusting for HPV DNA and antibodies.

## MATERIALS AND METHODS

The present study was conducted on women who previously participated in a Japanese case–control study of CIN. The details about this case–control study have been provided elsewhere (Yoshikawa *et al*, 1999). Among a total of 167 pairs, serum samples from 26 cases and 58 controls were not available for HPV capsid serology. Therefore, the present analysis was restricted to 250 subjects consisting of 141 cases (80 CIN I, 34 CIN II and 27 CIN III) and 109 controls that were tested for both cervical HPV DNA and serum HPV antibodies. The distributions of study variables in the excluded/included cases and controls were similar. We examined HPV DNA in cervical samples by polymerase chain reaction (PCR) using consensus primers for the HPV L1 region (Yoshikawa *et al*, 1999). Detection of IgG antibodies to HPV16, 52 and 58, the most frequently detected HPV types in Japan, was performed by enzyme-linked immunosorbent assay (ELISA) using purified L1-capsids (virus-like particles (VLPs)) as antigens (Matsumoto *et al*, 2003). In addition, the level of IgG antibodies to *Chlamydia trachomatis* was determined by using an enzyme immunoassay (EIA) kit (Thermo Labsystems, Vantaa, Finland) that does not detect antibody to *Chlamydia pneumoniae*. The adjusted odds ratios (ORs) and 95% confidence intervals (CIs) were estimated by a logistic regression analysis. The analysis was carried out using JMP 4.0J statistics package (SAS Institute, Cary, NC, USA). The *P*-values obtained in all tests were considered significant at <0.05.

## RESULTS

Human papillomavirus status among cases and controls was determined by using HPV DNA testing and HPV capsid serology (Table 1

**Table 1:** Human papillomavirus status among cases and controls

**HPV status**			
**DNA**	**Antibodies^a^**	**Case (*n*=141)**	**Control (*n*=109)**
—	—	21 (15%)	65 (60%)
—	+	15 (11%)	28 (26%)
+	—	44 (31%)	11 (10%)
+	+	61 (43%)	5 (4%)

a IgG antibodies to any type of HPV16/52/58 L1-capsids.

). We reconfirmed that HPV DNA positivity and HPV seropositivity were strongly associated with CIN development (*P*<0.0001 and =0.0002, respectively), although the relative risk for CIN was considerably different between HPV DNA positive and seropositive women (17.9 and 2.7, respectively) (Table 2

**Table 2:** Human papillomavirus infections and the estimated risks for CIN

	**Case (*n*=141)**	**Control (*n*=109)**	**OR (95% CI)^a^**
HPV DNA			
Negative	36 (26%)	93 (85%)	1.0
Positive	105 (74%)	16 (15%)	17.9 (9.5–36.0)

HPV antibodies^b^			
Negative	65 (46%)	76 (70%)	1.0
Positive	76 (54%)	33 (30%)	2.7 (1.6–4.6)

a odds ratios and 95% confidence intervals adjusted for age.

b IgG antibodies to any type of HPV16/52/58 L1-capsids.

). The estimated risks for CIN development in relation to various epidemiological factors are shown in Table 3

**Table 3:** Estimated risks for CIN associated with epidemiological cofactors

**Variables**	**Number of cases/controls**	**Crude OR^a^ (95% CI)^b^**	**OR adjusted for HPV DNA and Age (95% CI)**	**OR adjusted for HPV Ab^c^ and Age (95% CI)**
Marital status				
Never married	8/14	1.0	1.0	1.0
Separated/widowed	12/11	1.9 (0.6–6.3)	0.5 (0.1–2.7)	1.3 (0.3–5.1)
Married	121/84	2.5 (1.0–6.1)	3.9 (1.2–13.3)	2.8 (1.1–7.4)
		*P*=0.04	*P*=0.04	*P*=0.04

Parity				
0	12/18	1.0	1.0	1.0
1–2	78/66	1.7 (0.8–3.9)	2.0 (0.7–5.9)	1.3 (0.6–2.9)
3+	50/24	3.1 (1.3–7.4)	4.7 (1.4–15.7)	3.5 (1.4–9.1)
		*P* for trend=0.01	*P*=0.01	*P*=0.03

Age at first pregnancy (years)				
–23	63/26	2.0 (1.1–3.7)	3.5 (1.4–9.2)	3.5 (1.6–7.9)
24–26	37/38	1.0	1.0	1.0
27+	31/26	1.1 (0.6–2.1)	0.9 (0.3–2.6)	0.7 (0.3–1.7)
		*P* for trend=0.03	*P*=0.01	*P*=0.01

Cigarette smoking				
Never	94/78	1.0	1.0	1.0
Past	13/14	0.8 (0.3–1.7)	0.3 (0.1–1.1)	0.4 (0.1–1.2)
Current	34/17	1.7 (0.9–3.2)	3.0 (0.9–9.9)	3.2 (1.2–8.8)
		*P*=0.13	*P*=0.09	*P*=0.04

IgG antibody to *C.* *trachomatis*				
Negative	109/98	1.0	1.0	1.0
Positive	32/11	2.6 (1.3–5.4)	1.7 (0.7–4.3)	2.7 (1.3–6.0)
		*P*=0.01	*P*=0.26	*P*=0.01

Use of oral contraceptives				
Never	130/98	1.0	1.0	1.0
Ever	7/9	0.6 (0.2–1.6)	0.9 (0.3–2.8)	0.6 (0.2–1.4)
		*P*=0.30	*P*=0.94	*P*=0.18

Age at first intercourse (years)				
–23	111/74	1.9 (1.1–3.4)	1.8 (0.9–3.8)	1.8 (0.9–3.3)
24+	27/34	1.0	1.0	1.0
		*P*=0.03	*P*=0.14	*P*=0.10

Lifetime number of sexual partners				
0–1	65/64	1.0	1.0	1.0
2–3	42/28	1.5 (0.8–2.7)	0.7 (0.2–1.8)	1.1 (0.5–2.4)
4+	34/17	2.0 (1.0–3.9)	2.2 (0.7–7.0)	2.0 (0.8–5.3)
		*P* for trend=0.04	*P*=0.41	*P*=0.15

a Odds ratios.

b 95% confidence intervals.

c IgG antibodies to any type of HPV16/52/58 L1-capsids.

. In the present study, the adjusted analysis for HPV DNA alone gave the same results as our previous study (Yoshikawa *et al*, 1999). Marriage, multiparity and age at first pregnancy were found to be significant risk factors for CIN development in the crude and adjusted analyses. Sexual behaviour, including age at first intercourse and lifetime number of sexual partners, was significantly associated with an increased risk of CIN in the crude analysis, but the significance disappeared in both adjusted analyses. Current smoking and *C. trachomatis* infection were revealed as significant cofactors for CIN development after adjusting for HPV antibodies, although not after adjusting for HPV DNA. We found no association between the use of oral contraceptives (OC) and CIN development because of the low prevalence of OC users among study subjects. The effect of smoking on CIN development did not appear to vary between HPV-positive and HPV-negative subjects, while the effect of *C. trachomatis* infection was stronger among the former.

## DISCUSSION

With regard to smoking and *C. trachomatis* infection, the analysis adjusted for HPV antibodies gave different results from the analysis adjusted for HPV DNA. One explanation for this difference is that the antibody adjustment may reflect residual confounding by HPV, since only 54% of CIN cases were seropositive for HPV16/52/58 due to the limited sensitivity and high type-specificity of HPV capsid serology (Matsumoto *et al*, 2003). Another reasonable explanation is that smoking and *C. trachomatis* infection may be significant cofactors associated with persistent HPV infections. In controls, HPV DNA positivity was much lower than seropositivity (15 *vs* 30%, respectively). Since HPV DNA testing at a single time point cannot identify past infections, the analysis based on HPV DNA may miss cofactors determining whether HPV infection is cleared or becomes persistent. In fact, a very recent study reported that smoking is associated with a reduced probability of clearing oncogenic HPV infections (Giuliano *et al*, 2002). Associations of smoking and *C. trachomatis* infection with persistent HPV infections may be supported by several studies suggesting the modulation of host immunity by smoking (Barton *et al*, 1988) and chlamydial infection (Zhong *et al*, 1999).

Although the DNA adjustment enhanced the CIN risk among current smokers as well as the antibody adjustment, the association of smoking with an increased risk of CIN was not statistically significant in the DNA-based analysis. This may be due to the small numbers in the statistical analysis.

Unlike our previous study based on HPV DNA (Yoshikawa *et al*, 1999), the present study has shown that smoking and *C. trachomatis* infection are significant cofactors for CIN development after adjustment for HPV antibodies. Simi-larly, associations of smoking and *C. trachomatis* infection with cervical neoplasia have been consistent in case–control studies employing HPV capsid serology (Dillner *et al*, 1997; Olsen *et al*, 1998; Kjellberg *et al*, 2000; Koskela *et al*, 2000; Anttila *et al*, 2002), but inconsistent in HPV DNA-based studies (Schiffman *et al*, 1993; de Sanjosé *et al*, 1994; Eluf-Neto *et al*, 1994; Olsen *et al*, 1995; Ferrera *et al*, 1997). Our observation suggests that epidemiological studies relying on a single assessment of HPV status should be interpreted with caution. To fully evaluate the role of epidemiological cofactors in HPV-related carcinogenesis, allowance for both HPV DNA and antibodies may be necessary.
